# Supplementary material for: Differential analysis of mean blood glucose levels from venous and fingertip in predicting 30-day mortality among ICU patients with severe trauma: A retrospective study utilizing the MIMIC-IV database
Source: PLoS One. 2026 Feb 23;21(2):e0343401. doi: 10.1371/journal.pone.0343401 (PMC12928430; doi:10.1371/journal.pone.0343401)
Supplement: S1 Table — VMBG: mean blood glucose of venous. FMBG: mean blood glucose of fingertip. (DOCX) [file pone.0343401.s001.docx]

**Supplementary Table 1** Comparison of VMBG and FMBG between mortality group and survival group at various time intervals

| **Variables** | **Overall** | **30-day survial** | **30-day mortality** | **p** |
| --- | --- | --- | --- | --- |
| N | 2699 | 2361 | 338 |  |
| VMBG within 24 hours (mg/dL) | 134.33 [113.00, 163.33] | 132.50 [112.00, 161.00] | 149.00 [126.75, 180.67] | <0.001 |
| VMBG within 2 days (mg/dL) | 132.00 [113.33, 155.50] | 130.00 [112.33, 153.00] | 148.25 [126.06, 175.78] | <0.001 |
| VMBG within 3 days (mg/dL) | 129.00 [112.50, 153.00] | 127.33 [111.00, 149.25] | 147.10 [127.25, 174.50] | <0.001 |
| VMBG within 5 days (mg/dL) | 127.00 [111.50, 150.77] | 125.00 [110.50, 146.33] | 148.33 [126.43, 172.24] | <0.001 |
| VMBG within 10 days (mg/dL) | 126.22 [111.32, 149.17] | 123.67 [110.20, 144.44] | 147.42 [128.47, 175.54] | <0.001 |
| VMBG within 20 days (mg/dL) | 125.26 [110.81, 147.75] | 123.00 [109.52, 143.29] | 146.56 [127.70, 175.19] | <0.001 |
| FMBG within 24 hours (mg/dL) | 133.33 [112.42, 160.00] | 131.50 [111.00, 157.00] | 149.00 [124.58, 178.38] | <0.001 |
| FMBG within 2 days (mg/dL) | 133.40 [113.83, 156.50] | 131.40 [113.00, 154.00] | 149.10 [124.90, 172.45] | <0.001 |
| FMBG within 3 days (mg/dL) | 133.30 [114.67, 155.41] | 131.20 [113.75, 152.78] | 149.50 [126.93, 173.55] | <0.001 |
| FMBG within 5 days (mg/dL) | 133.00 [115.50, 156.00] | 131.29 [114.67, 153.31] | 150.00 [127.95, 173.71] | <0.001 |
| FMBG within 10 days (mg/dL) | 133.67 [116.74, 156.65] | 132.00 [115.38, 154.00] | 150.25 [129.50, 176.06] | <0.001 |
| FMBG within 20 days (mg/dL) | 133.60 [116.93, 156.47] | 131.82 [115.50, 154.00] | 150.66 [129.50, 174.64] | <0.001 |

VMBG: mean blood glucose of venous. FMBG: mean blood glucose of fingertip.
