# Supplementary material for: Differential analysis of mean blood glucose levels from venous and fingertip in predicting 30-day mortality among ICU patients with severe trauma: A retrospective study utilizing the MIMIC-IV database
Source: PLoS One. 2026 Feb 23;21(2):e0343401. doi: 10.1371/journal.pone.0343401 (PMC12928430; doi:10.1371/journal.pone.0343401)
Supplement: S2 Table — VMBG: mean blood glucose of venous. FMBG: mean blood glucose of fingertip. Adjusted for sex, age, race, comorbidity index, cerebrovascular disease, liver disease, chronic pulmonary disease, diabetes, congestive heart failure, cancer, renal disease, CRRT, ventilation, insulin, transfusion, SOFA, GCS, AKI stage, SAPSⅡ, APSⅢ, OASIS. (DOCX) [file pone.0343401.s002.docx]

**Supplementary Table 2** Multivariate COX regression analysis of VMBG and FMBG at different time intervals

| **Time Intervals** | **VMBG** | |  |  | **FMBG** | |  |  |
| --- | --- | --- | --- | --- | --- | --- | --- | --- |
|  | **HR(95%CI)** | **P** | **C-index** | **Brier Score** | **HR(95%CI)** | **P** | **C-index** | **Brier Score** |
| within 24 hours (mg/dL) | 1.003(1.001,1.006) | 0.006 | 0.811 | 0.088 | 1.004(1.002,1.007) | 0.002 | 0.812 | 0.088 |
| within 2 days (mg/dL) | 1.007(1.004,1.010) | ＜0.001 | 0.815 | 0.088 | 1.007(1.003,1.010) | ＜0.001 | 0.814 | 0.088 |
| within 3 days (mg/dL) | 1.010(1.007,1.013) | ＜0.001 | 0.819 | 0.087 | 1.009(1.005,1.012) | ＜0.001 | 0.817 | 0.088 |
| within 5 days (mg/dL) | 1.013(1.009,1.016) | ＜0.001 | 0.822 | 0.086 | 1.009(1.005,1.013) | ＜0.001 | 0.816 | 0.087 |
| within 10 days (mg/dL) | 1.016(1.012,1.020) | ＜0.001 | 0.828 | 0.085 | 1.009(1.006,1.013) | ＜0.001 | 0.816 | 0.087 |
| within 20 days (mg/dL) | 1.019(1.015,1.022) | ＜0.001 | 0.832 | 0.083 | 1.009(1.005,1.013) | ＜0.001 | 0.816 | 0.087 |
| within 30 days (mg/dL) | 1.019(1.016,1.023) | ＜0.001 | 0.833 | 0.083 | 1.009(1.006,1.013) | ＜0.001 | 0.816 | 0.087 |

VMBG: mean blood glucose of venous. FMBG: mean blood glucose of fingertip. Adjusted for sex, age, race, comorbidity index, cerebrovascular disease, liver disease, chronic pulmonary disease, diabetes, congestive heart failure, cancer, renal disease, CRRT, ventilation, insulin, transfusion, SOFA, GCS, AKI stage, SAPSⅡ, APSⅢ, OASIS
