# Supplementary material for: Differential analysis of mean blood glucose levels from venous and fingertip in predicting 30-day mortality among ICU patients with severe trauma: A retrospective study utilizing the MIMIC-IV database
Source: PLoS One. 2026 Feb 23;21(2):e0343401. doi: 10.1371/journal.pone.0343401 (PMC12928430; doi:10.1371/journal.pone.0343401)
Supplement: S3 Table — (DOCX) [file pone.0343401.s003.docx]

**Supplementary Table 3** The comparison between VMBG, FMBG and 30-day mortality of the landmark cohorts

| **Variables** | **Overall** | **30-day survial** | **30-day mortality** | **p** |
| --- | --- | --- | --- | --- |
| *Landmark at tine point of 2 days* | |  |  |  |
| N | 2634 | 2332 | 302 |  |
| VMBG | 132.00 [113.33, 155.00] | 130.00 [112.50, 153.00] | 147.06 [124.58, 170.69] | <0.001 |
| FMBG | 133.40 [114.00, 156.11] | 131.50 [113.31, 154.00] | 148.47 [124.42, 170.13] | <0.001 |
| *Landmark at tine point of 3 days* | |  |  |  |
| N | 2516 | 2242 | 274 |  |
| VMBG | 129.00 [113.00, 152.50] | 127.67 [112.23, 150.00] | 145.17 [125.04, 164.88] | <0.001 |
| FMBG | 133.33 [115.00, 154.69] | 131.61 [114.10, 152.89] | 146.14 [124.88, 166.69] | <0.001 |
