# Supplementary material for: Differential analysis of mean blood glucose levels from venous and fingertip in predicting 30-day mortality among ICU patients with severe trauma: A retrospective study utilizing the MIMIC-IV database
Source: PLoS One. 2026 Feb 23;21(2):e0343401. doi: 10.1371/journal.pone.0343401 (PMC12928430; doi:10.1371/journal.pone.0343401)
Supplement: S4 Table — Adjusted for sex, age, race, comorbidity index, cerebrovascular disease, liver disease, chronic pulmonary disease, diabetes, congestive heart failure, cancer, renal disease, CRRT, ventilation, insulin, transfusion, SOFA, GCS, AKI stage, SAPSⅡ, APSⅢ, OASIS. (DOCX) [file pone.0343401.s004.docx]

**Supplementary Table 4** Multivariate Cox regression analysis of VMBG, FMBG and 30-day mortality of the Landmark cohorts

| **Models** | **HR(95%CI)** | **P** | **C-index** | **Brier Score** |
| --- | --- | --- | --- | --- |
| *Landmark at tine point of 2 days* | | |  |  |
| VMBG | 1.004(1.001,1.008) | 0.013 | 0.813 | 0.083 |
| FMBG | 1.004(1.001,1.008) | 0.027 | 0.813 | 0.083 |
| *Landmark at tine point of 3 days* | | |  |  |
| VMBG | 1.006(1.003,1.010) | ＜0.001 | 0.812 | 0.080 |
| FMBG | 1.005(1.001,1.009) | 0.021 | 0.811 | 0.080 |

Adjusted for sex, age, race, comorbidity index, cerebrovascular disease, liver disease, chronic pulmonary disease, diabetes, congestive heart failure, cancer, renal disease, CRRT, ventilation, insulin, transfusion, SOFA, GCS, AKI stage, SAPSⅡ, APSⅢ, OASIS.
