# Supplementary material for: Differential analysis of mean blood glucose levels from venous and fingertip in predicting 30-day mortality among ICU patients with severe trauma: A retrospective study utilizing the MIMIC-IV database
Source: PLoS One. 2026 Feb 23;21(2):e0343401. doi: 10.1371/journal.pone.0343401 (PMC12928430; doi:10.1371/journal.pone.0343401)
Supplement: S5 Table — VMBG: mean blood glucose of venous within 30 days. FMBG: mean blood glucose of fingertip within 30 days. VGV: glycemic variability of venous. FGV: glycemic variability of fingertip. SBP: systolic blood pressure. DBP: diastolic blood pressure. MAP: mean arterial pressure. WBC: white blood cell. RBC: red blood cell. RDW: red cell distribution width. INR: international normalized ratio. PT: prothrombin time. PTT: partial thromboplastin time. GCS: Glasgow Coma Scale. SOFA: Sequential Organ Failure Assessment. SAPS Ⅱ: Simplified Acute Physiology Scores Ⅱ. APS Ⅲ: Acute Physiology Score Ⅲ. OASIS: Oxford Acute Severity of Illness Score. AKI stage: acute kidney injury stage. CRRT: continuous renal replacement therapy. (DOCX) [file pone.0343401.s005.docx]

**Supplementary Table 5** Baseline characteristics of the complete dataset

| **Variables** | **Overall** | **30-day survial** | **30-day mortality** | **p** | **SMD** |
| --- | --- | --- | --- | --- | --- |
| N | 2501 | 2183 | 318 |  |  |
| Age (year) | 64.62 [48.23, 78.22] | 63.03 [46.18, 76.38] | 76.89 [62.95, 84.62] | <0.001 | 0.605 |
| Male (%) | 1656 (66.2) | 1469 (67.3) | 187 ( 58.8) | 0.003 | 0.177 |
| Weight (kg) | 77.40 [65.40, 91.70] | 78.00 [66.18, 92.30] | 73.05 [60.83, 87.50] | <0.001 | 0.221 |
| Race (%) |  |  |  | <0.001 | 0.356 |
| Other | 724 (28.9) | 608 (27.9) | 116 ( 36.5) |  |  |
| Hispanic | 109 ( 4.4) | 106 ( 4.9) | 3 ( 0.9) |  |  |
| Black | 124 ( 5.0) | 119 ( 5.5) | 5 ( 1.6) |  |  |
| Asian | 49 ( 2.0) | 45 ( 2.1) | 4 ( 1.3) |  |  |
| White | 1495 (59.8) | 1305 (59.8) | 190 ( 59.7) |  |  |
| *Vital signs* |  |  |  |  |  |
| Heart rate (bpm) | 83.31 [72.36, 95.89] | 83.42 [72.33, 95.86] | 82.13 [72.65, 96.07] | 0.94 | 0.003 |
| SBP (mmHg) | 123.03 [112.38, 133.31] | 123.04 [112.39, 133.31] | 122.62 [112.46, 133.37] | 0.778 | 0.03 |
| DBP (mmHg) | 64.07 [57.83, 71.92] | 64.55 [58.30, 72.22] | 60.98 [54.63, 68.47] | <0.001 | 0.394 |
| MBP (mmHg) | 80.53 [74.11, 87.62] | 80.71 [74.24, 87.96] | 79.45 [73.35, 85.57] | 0.006 | 0.213 |
| Resp rate (bpm) | 17.98 [16.25, 20.10] | 17.87 [16.19, 19.96] | 18.74 [16.94, 21.68] | <0.001 | 0.387 |
| Temperature (℃) | 37.00 [36.75, 37.32] | 37.01 [36.76, 37.31] | 36.97 [36.64, 37.37] | 0.183 | 0.137 |
| Spo2 (%) | 97.78 [96.30, 99.06] | 97.69 [96.27, 99.00] | 98.45 [96.76, 99.50] | <0.001 | 0.194 |
| *Scoring systems* |  |  |  |  |  |
| GCS | 14.00 [13.00, 15.00] | 14.00 [13.00, 15.00] | 15.00 [11.00, 15.00] | 0.044 | 0.304 |
| SOFA | 3.00 [2.00, 5.00] | 3.00 [2.00, 5.00] | 5.00 [3.00, 8.00] | <0.001 | 0.773 |
| SAPSⅡ | 32.00 [24.00, 40.00] | 31.00 [23.00, 38.00] | 42.00 [34.25, 50.00] | <0.001 | 1.077 |
| APSⅢ | 36.00 [28.00, 48.00] | 35.00 [27.00, 45.00] | 49.00 [37.00, 61.75] | <0.001 | 0.804 |
| OASIS | 32.00 [27.00, 37.00] | 31.00 [26.00, 36.00] | 36.00 [32.00, 42.00] | <0.001 | 0.778 |
| AKI Stage (%) |  |  |  | <0.001 | 0.483 |
| 0 | 616 (24.6) | 573 (26.2) | 43 ( 13.5) |  |  |
| 1 | 464 (18.6) | 417 (19.1) | 47 ( 14.8) |  |  |
| 2 | 1042 (41.7) | 908 (41.6) | 134 ( 42.1) |  |  |
| 3 | 379 (15.2) | 285 (13.1) | 94 ( 29.6) |  |  |
| *Laboratory parameters* |  |  |  |  |  |
| VMBG (mg/dL) | 124.61 [110.90, 147.00] | 122.50 [109.51, 142.15] | 146.56 [127.97, 176.50] | <0.001 | 0.718 |
| FMBG (mg/dL) | 133.60 [117.00, 156.23] | 131.67 [115.81, 153.04] | 151.00 [130.00, 175.69] | <0.001 | 0.476 |
| Hematocrit (%) | 33.90 [29.62, 37.85] | 34.10 [30.00, 37.95] | 31.88 [27.84, 36.16] | <0.001 | 0.335 |
| Hemoglobin (g/dL) | 11.45 [9.85, 12.75] | 11.55 [10.00, 12.85] | 10.63 [9.11, 11.89] | <0.001 | 0.405 |
| Platelets (10^9/L) | 194.00 [149.14, 241.00] | 196.50 [152.31, 243.29] | 173.17 [130.25, 218.83] | <0.001 | 0.269 |
| WBC (10^9/L) | 11.33 [8.70, 14.40] | 11.24 [8.65, 14.25] | 12.00 [8.96, 15.56] | 0.008 | 0.192 |
| RBC (10^12/L) | 3.62 [3.15, 4.09] | 3.66 [3.19, 4.11] | 3.42 [2.96, 3.86] | <0.001 | 0.333 |
| RDW (%) | 13.90 [13.17, 15.00] | 13.80 [13.10, 14.83] | 14.60 [13.56, 15.90] | <0.001 | 0.432 |
| Anion gap (mmol/L) | 14.00 [12.00, 16.00] | 14.00 [12.00, 16.00] | 15.00 [12.69, 16.75] | <0.001 | 0.282 |
| Bicarbonate (mmol/L) | 23.00 [21.00, 25.33] | 23.33 [21.00, 25.50] | 22.00 [19.50, 24.50] | <0.001 | 0.345 |
| Bun (mg/dL) | 15.75 [11.67, 22.00] | 15.33 [11.33, 21.00] | 19.63 [15.00, 30.00] | <0.001 | 0.482 |
| Calcium (mmol/L) | 8.35 [7.90, 8.78] | 8.35 [7.90, 8.75] | 8.40 [7.95, 8.90] | 0.116 | 0.088 |
| Chloride (mmol/L) | 104.50 [101.50, 107.60] | 104.50 [101.50, 107.50] | 105.00 [101.50, 109.00] | 0.029 | 0.167 |
| Creatinine (mg/dL) | 0.90 [0.70, 1.10] | 0.87 [0.70, 1.10] | 1.00 [0.79, 1.40] | <0.001 | 0.27 |
| Sodium (mmol/L) | 139.00 [137.00, 141.25] | 139.00 [137.00, 141.00] | 139.50 [137.06, 142.33] | 0.002 | 0.226 |
| Potassium (mmol/L) | 4.10 [3.80, 4.45] | 4.10 [3.80, 4.45] | 4.16 [3.80, 4.50] | 0.203 | 0.088 |
| INR | 1.15 [1.07, 1.30] | 1.13 [1.05, 1.25] | 1.25 [1.10, 1.44] | <0.001 | 0.402 |
| PT | 12.80 [11.73, 14.20] | 12.70 [11.70, 14.00] | 13.70 [12.34, 15.69] | <0.001 | 0.388 |
| PTT | 27.50 [25.20, 30.67] | 27.30 [25.10, 30.38] | 28.90 [26.34, 33.18] | <0.001 | 0.33 |
| *Comorbidities* |  |  |  |  |  |
| Comorbidity index | 4.00 [2.00, 6.00] | 4.00 [2.00, 6.00] | 6.00 [4.00, 8.00] | <0.001 | 0.659 |
| Congestive heart failure (%) | 314 (12.6) | 249 (11.4) | 65 ( 20.4) | <0.001 | 0.249 |
| Cerebrovascular disease (%) | 235 ( 9.4) | 188 ( 8.6) | 47 ( 14.8) | 0.001 | 0.193 |
| Chronic pulmonary disease (%) | 393 (15.7) | 336 (15.4) | 57 ( 17.9) | 0.281 | 0.068 |
| Renal disease (%) | 278 (11.1) | 217 ( 9.9) | 61 ( 19.2) | <0.001 | 0.264 |
| Liver disease (%) | 178 ( 7.1) | 138 ( 6.3) | 40 ( 12.6) | <0.001 | 0.215 |
| Cancer (%) | 93 ( 3.7) | 72 ( 3.3) | 21 ( 6.6) | 0.006 | 0.153 |
| Diabetes (%) | 644 (25.7) | 536 (24.6) | 108 ( 34.0) | <0.001 | 0.208 |
| *Treatment* |  |  |  |  |  |
| CRRT (%) | 26 ( 1.0) | 10 ( 0.5) | 16 ( 5.0) | <0.001 | 0.283 |
| Ventilation (%) | 1385 (55.4) | 1137 (52.1) | 248 ( 78.0) | <0.001 | 0.564 |
| Transfusion (%) | 904 (36.1) | 771 (35.3) | 133 ( 41.8) | 0.028 | 0.134 |
| Insulin (%) | 1310 (52.4) | 1105 (50.6) | 205 ( 64.5) | <0.001 | 0.283 |
| *Outcomes* |  |  |  |  |  |
| Los hospital (days) | 8.92 [5.36, 16.32] | 9.33 [5.57, 16.86] | 7.18 [3.94, 11.65] | <0.001 | 0.467 |
| Los icu (days) | 3.23 [1.87, 6.80] | 3.06 [1.83, 6.34] | 4.86 [2.58, 8.46] | <0.001 | 0.144 |
| In-hospital mortality (%) | 263 (10.5) | 12 ( 0.5) | 251 ( 78.9) | <0.001 | 2.675 |
| 90-day mortality (%) | 402 (16.1) | 84 ( 3.8) | 318 (100.0) | <0.001 | 7.069 |
| 180-day mortality (%) | 469 (18.8) | 151 ( 6.9) | 318 (100.0) | <0.001 | 5.188 |
| 360-day mortality (%) | 540 (21.6) | 222 (10.2) | 318 (100.0) | <0.001 | 4.203 |

VMBG: mean blood glucose of venous within 30 days. FMBG: mean blood glucose of fingertip within 30 days. VGV: glycemic variability of venous. FGV: glycemic variability of fingertip. SBP: systolic blood pressure. DBP: diastolic blood pressure. MAP: mean arterial pressure. WBC: white blood cell. RBC: red blood cell. RDW: red cell distribution width. INR: international normalized ratio. PT: prothrombin time. PTT: partial thromboplastin time. GCS: Glasgow Coma Scale. SOFA: Sequential Organ Failure Assessment. SAPS Ⅱ: Simplified Acute Physiology Scores Ⅱ. APS Ⅲ: Acute Physiology Score Ⅲ. OASIS: Oxford Acute Severity of Illness Score. AKI stage: acute kidney injury stage. CRRT: continuous renal replacement therapy.
