# Supplementary material for: Differential analysis of mean blood glucose levels from venous and fingertip in predicting 30-day mortality among ICU patients with severe trauma: A retrospective study utilizing the MIMIC-IV database
Source: PLoS One. 2026 Feb 23;21(2):e0343401. doi: 10.1371/journal.pone.0343401 (PMC12928430; doi:10.1371/journal.pone.0343401)
Supplement: S6 Table — VMBG: mean blood glucose of venous. FMBG: mean blood glucose of fingertip. Model1: adjusted for sex, age, race. Model2: adjusted for sex, age, race, comorbidity index, cerebrovascular disease, liver disease, chronic pulmonary disease, diabetes, congestive heart failure, cancer, renal disease. Model3: adjusted for sex, age, race, comorbidity index, cerebrovascular disease, liver disease, chronic pulmonary disease, diabetes, congestive heart failure, cancer, renal disease, CRRT, ventilation, insulin, transfusion, SOFA, GCS, AKI stage, SAPSⅡ, APSⅢ, OASIS. (DOCX) [file pone.0343401.s006.docx]

**Supplementary Table 6** COX regression analysis of VMBG and FMBG in the complete dataset

| **Variable** | **Crude** |  | **Model 1** |  | **Model 2** |  | **Model 3** |  |
| --- | --- | --- | --- | --- | --- | --- | --- | --- |
|  | **HR(95%CI)** | **P** | **HR(95%CI)** | **P** | **HR(95%CI)** | **P** | **HR(95%CI)** | **P** |
| **VMBG** |  |  |  |  |  |  |  |  |
| ＜88.1mg/dl | 3.504(1.604,7.656) | 0.002 | 2.848(1.302,6.231) | 0.009 | 2.695(1.228,5.913) | 0.013 | 3.274(1.476,7.263) | 0.004 |
| 88.1-125.4mg/dl | 1(reference) | - | 1(reference) | - | 1(reference) | - | 1(reference) | - |
| ≥125.4mg/dl | 4.349(3.293,5.744) | ＜0.001 | 3.723(2.814,4.924) | ＜0.001 | 4.102(3.063,5.494) | ＜0.001 | 3.241(2.378,4.418) | ＜0.001 |
| continuous | 1.018(1.015,1.020) | ＜0.001 | 1.017(1.014,1.020) | ＜0.001 | 1.022(1.019,1.025) | ＜0.001 | 1.020(1.166,1.024) | ＜0.001 |
| **FMBG** |  |  |  |  |  |  |  |  |
| ＜95.4mg/dl | 1.735(1.028,2.926) | 0.039 | 1.948(1.153,3.289) | 0.013 | 1.822(1.077,3.083) | 0.025 | 1.950(1.142,3.331) | 0.014 |
| 95.4-134.0mg/dl | 1(reference) | - | 1(reference) | - | 1(reference) | - | 1(reference) | - |
| ≥134.0mg/dl | 2.658(2.060,3.429) | ＜0.001 | 2.268(1.754,2.933) | ＜0.001 | 2.311(1.762,3.029) | ＜0.001 | 1.988(1.485,2.662) | ＜0.001 |
| continuous | 1.012(1.009,1.015) | ＜0.001 | 1.009(1.006,1.012) | ＜0.001 | 1.011(1.008,1.015) | ＜0.001 | 1.010(1.006,1.014) | ＜0.001 |

VMBG: mean blood glucose of venous. FMBG: mean blood glucose of fingertip. Model1: adjusted for sex, age, race. Model2: adjusted for sex, age, race, comorbidity index, cerebrovascular disease, liver disease, chronic pulmonary disease, diabetes, congestive heart failure, cancer, renal disease. Model3: adjusted for sex, age, race, comorbidity index, cerebrovascular disease, liver disease, chronic pulmonary disease, diabetes, congestive heart failure, cancer, renal disease, CRRT, ventilation, insulin, transfusion, SOFA, GCS, AKI stage, SAPSⅡ, APSⅢ, OASIS.
