# Supplementary material for: Differential analysis of mean blood glucose levels from venous and fingertip in predicting 30-day mortality among ICU patients with severe trauma: A retrospective study utilizing the MIMIC-IV database
Source: PLoS One. 2026 Feb 23;21(2):e0343401. doi: 10.1371/journal.pone.0343401 (PMC12928430; doi:10.1371/journal.pone.0343401)
Supplement: S7 Table — (DOCX) [file pone.0343401.s007.docx]

**Supplementary Table 7** The comparison between VMBG and FMBG within 30 days without trimming

| **Variables** | **Overall** | **30-day survial** | **30-day mortality** | **p** |
| --- | --- | --- | --- | --- |
| N | 2699 | 2361 | 338 |  |
| VMBG | 125.14 [110.84, 147.47] | 122.56 [109.50, 142.83] | 146.56 [127.62, 175.77] | <0.001 |
| FMBG | 133.60 [116.89, 156.40] | 131.82 [115.50, 153.83] | 150.66 [129.50, 174.64] | <0.001 |
