# Supplementary material for: Differential analysis of mean blood glucose levels from venous and fingertip in predicting 30-day mortality among ICU patients with severe trauma: A retrospective study utilizing the MIMIC-IV database
Source: PLoS One. 2026 Feb 23;21(2):e0343401. doi: 10.1371/journal.pone.0343401 (PMC12928430; doi:10.1371/journal.pone.0343401)
Supplement: S9 Table — VMBG: mean blood glucose of venous. FMBG: mean blood glucose of fingertip. SBP: systolic blood pressure. DBP: diastolic blood pressure. MAP: mean arterial pressure. WBC: white blood cell. RBC: red blood cell. RDW: red cell distribution width. INR: international normalized ratio. PT: prothrombin time. PTT: partial thromboplastin time. GCS: Glasgow Coma Scale. SOFA: Sequential Organ Failure Assessment. SAPS Ⅱ: Simplified Acute Physiology Scores Ⅱ. APS Ⅲ: Acute Physiology Score Ⅲ. OASIS: Oxford Acute Severity of Illness Score. AKI stage: acute kidney injury stage. CRRT: continuous renal replacement therapy. PSM: propensity score matching. (DOCX) [file pone.0343401.s009.docx]

**Supplementary Table 9** Baseline characteristics after PSM

| **Variables** | **Overall** | **30-day survial** | **30-day mortality** | **p** | **SMD** |
| --- | --- | --- | --- | --- | --- |
| N | 798 | 503 | 295 |  |  |
| Age (year) | 75.93 [63.22, 83.95] | 75.28 [63.66, 83.84] | 76.70 [62.73, 84.10] | 0.628 | -0.025 |
| Male (%) | 520 (62.6) | 335 (64.1) | 185 ( 60.1) | 0.283 | -0.036 |
| Weight (kg) | 73.40 [62.20, 87.97] | 73.40 [62.42, 88.00] | 73.37 [61.70, 87.96] | 0.694 | 0.026 |
| Race (%) |  |  |  | <0.001 | -0.044 |
| Other | 257 (30.9) | 144 (27.5) | 113 ( 36.7) |  |  |
| Hispanic | 28 ( 3.4) | 25 ( 4.8) | 3 ( 1.0) |  |  |
| Black | 44 ( 5.3) | 40 ( 7.6) | 4 ( 1.3) |  |  |
| Asian | 17 ( 2.0) | 13 ( 2.5) | 4 ( 1.3) |  |  |
| White | 485 (58.4) | 301 (57.6) | 184 ( 59.7) |  |  |
| *Vital signs* |  |  |  |  |  |
| Heart rate (bpm) | 82.19 [71.96, 94.55] | 82.63 [71.56, 94.27] | 81.71 [72.31, 95.14] | 0.760 | 0.003 |
| SBP (mmHg) | 123.35 [113.05, 134.02] | 123.28 [112.97, 134.10] | 123.68 [113.26, 133.58] | 0.814 | 0.025 |
| DBP (mmHg) | 61.60 [55.40, 68.64] | 61.69 [55.68, 68.62] | 61.19 [55.24, 68.76] | 0.508 | -0.024 |
| MBP (mmHg) | 79.52 [73.63, 86.54] | 79.07 [73.42, 86.81] | 79.62 [74.17, 85.79] | 0.724 | 0.002 |
| Resp rate (bpm) | 18.73 [16.89, 21.33] | 18.83 [16.94, 21.35] | 18.66 [16.86, 21.29] | 0.951 | 0.014 |
| Temperature (℃) | 37.00 [36.71, 37.38] | 37.02 [36.73, 37.39] | 36.97 [36.66, 37.38] | 0.425 | -0.033 |
| Spo2 (%) | 98.19 [96.75, 99.31] | 98.08 [96.80, 99.16] | 98.46 [96.66, 99.50] | 0.112 | 0.013 |
| *Scoring systems* |  |  |  |  |  |
| GCS | 14.00 [11.00, 15.00] | 14.00 [11.00, 15.00] | 15.00 [11.00, 15.00] | 0.596 | 0.001 |
| SOFA | 4.00 [3.00, 7.00] | 4.00 [3.00, 6.00] | 5.00 [3.00, 7.00] | 0.026 | 0.047 |
| SAPSⅡ | 40.00 [33.00, 47.00] | 40.00 [33.00, 46.00] | 40.00 [34.00, 49.00] | 0.049 | 0.015 |
| APSⅢ | 45.00 [34.00, 58.00] | 43.00 [33.00, 58.00] | 47.00 [36.00, 58.00] | 0.036 | 0.037 |
| OASIS | 35.00 [31.00, 40.00] | 35.00 [31.00, 40.00] | 36.00 [31.00, 41.00] | 0.115 | 0.014 |
| AKI Stage (%) |  |  |  | 0.553 | 0.007 |
| 0 | 126 (15.2) | 81 (15.5) | 45 ( 14.6) |  |  |
| 1 | 124 (14.9) | 78 (14.9) | 46 ( 14.9) |  |  |
| 2 | 377 (45.4) | 244 (46.7) | 133 ( 43.2) |  |  |
| 3 | 204 (24.5) | 120 (22.9) | 84 ( 27.3) |  |  |
| *Laboratory parameters* |  |  |  |  |  |
| Hematocrit (%) | 32.50 [28.65, 36.33] | 32.58 [29.01, 36.50] | 32.34 [28.47, 36.25] | 0.397 | 0.005 |
| Hemoglobin (g/dL) | 10.75 [9.40, 12.10] | 10.77 [9.44, 12.20] | 10.70 [9.26, 11.88] | 0.320 | 0.004 |
| Platelets (10^9/L) | 175.75 [135.27, 228.75] | 175.67 [136.00, 232.83] | 176.62 [134.94, 221.17] | 0.562 | -0.006 |
| WBC (10^9/L) | 11.70 [8.74, 15.09] | 11.55 [8.60, 14.75] | 12.00 [9.02, 15.39] | 0.174 | 0.064 |
| RBC (10^12/L) | 3.44 [3.04, 3.91] | 3.44 [3.07, 3.93] | 3.45 [2.99, 3.87] | 0.641 | 0.009 |
| RDW (%) | 14.45 [13.40, 15.68] | 14.40 [13.40, 15.58] | 14.57 [13.50, 15.90] | 0.232 | 0.003 |
| Anion gap (mmol/L) | 14.33 [12.33, 16.50] | 14.00 [12.00, 16.29] | 14.63 [12.65, 16.67] | 0.123 | 0.048 |
| Bicarbonate (mmol/L) | 23.00 [20.29, 25.00] | 23.00 [20.45, 25.12] | 22.50 [20.00, 24.69] | 0.057 | -0.086 |
| Bun (mg/dL) | 19.50 [14.21, 28.37] | 19.50 [14.00, 28.00] | 19.50 [14.93, 29.54] | 0.313 | 0.019 |
| Calcium (mmol/L) | 8.40 [8.00, 8.85] | 8.40 [8.00, 8.85] | 8.40 [7.97, 8.83] | 0.795 | -0.030 |
| Chloride (mmol/L) | 105.00 [102.00, 108.45] | 105.00 [102.29, 108.37] | 105.00 [101.94, 108.50] | 0.985 | 0.004 |
| Creatinine (mg/dL) | 0.97 [0.76, 1.40] | 0.95 [0.76, 1.36] | 0.98 [0.77, 1.42] | 0.382 | -0.013 |
| Sodium (mmol/L) | 139.75 [137.50, 142.00] | 140.00 [137.50, 142.00] | 139.50 [137.31, 142.33] | 0.662 | -0.015 |
| Potassium (mmol/L) | 4.10 [3.80, 4.50] | 4.10 [3.80, 4.50] | 4.15 [3.82, 4.50] | 0.589 | 0.015 |
| INR | 1.20 [1.10, 1.40] | 1.20 [1.10, 1.37] | 1.23 [1.10, 1.44] | 0.012 | 0.014 |
| PT | 13.30 [12.13, 15.39] | 13.13 [12.05, 15.12] | 13.57 [12.29, 15.58] | 0.028 | 0.018 |
| PTT | 28.20 [25.60, 31.88] | 27.97 [25.42, 31.18] | 28.70 [25.98, 32.71] | 0.026 | 0.047 |
| *Comorbidities* |  |  |  |  |  |
| Comorbidity index | 5.00 [3.00, 7.00] | 5.00 [3.00, 7.00] | 5.00 [3.00, 7.00] | 0.715 | -0.030 |
| Congestive heart failure (%) | 176 (21.2) | 111 (21.2) | 65 ( 21.1) | 1.000 | -0.015 |
| Cerebrovascular disease (%) | 112 (13.5) | 68 (13.0) | 44 ( 14.3) | 0.676 | 0.007 |
| Chronic pulmonary disease (%) | 143 (17.2) | 85 (16.3) | 58 ( 18.8) | 0.392 | 0.020 |
| Renal disease (%) | 161 (19.4) | 99 (18.9) | 62 ( 20.1) | 0.740 | -0.003 |
| Liver disease (%) | 92 (11.1) | 57 (10.9) | 35 ( 11.4) | 0.927 | -0.008 |
| Cancer (%) | 50 ( 6.0) | 31 ( 5.9) | 19 ( 6.2) | 1.000 | -0.002 |
| Diabetes (%) | 274 (33.0) | 169 (32.3) | 105 ( 34.1) | 0.653 | 0.013 |
| *Treatment* |  |  |  |  |  |
| CRRT (%) | 23 ( 2.8) | 10 ( 1.9) | 13 ( 4.2) | 0.082 | 0.018 |
| Ventilation (%) | 606 (72.9) | 374 (71.5) | 232 ( 75.3) | 0.265 | 0.010 |
| Transfusion (%) | 328 (39.5) | 205 (39.2) | 123 ( 39.9) | 0.891 | -0.005 |
| Insulin (%) | 532 (64.0) | 335 (64.1) | 197 ( 64.0) | 1.000 | -0.010 |

VMBG: mean blood glucose of venous. FMBG: mean blood glucose of fingertip. SBP: systolic blood pressure. DBP: diastolic blood pressure. MAP: mean arterial pressure. WBC: white blood cell. RBC: red blood cell. RDW: red cell distribution width. INR: international normalized ratio. PT: prothrombin time. PTT: partial thromboplastin time. GCS: Glasgow Coma Scale. SOFA: Sequential Organ Failure Assessment. SAPS Ⅱ: Simplified Acute Physiology Scores Ⅱ. APS Ⅲ: Acute Physiology Score Ⅲ. OASIS: Oxford Acute Severity of Illness Score. AKI stage: acute kidney injury stage. CRRT: continuous renal replacement therapy. PSM: propensity score matching.
