# Supplementary figures and images for: Differential analysis of mean blood glucose levels from venous and fingertip in predicting 30-day mortality among ICU patients with severe trauma: A retrospective study utilizing the MIMIC-IV database
Source: PLoS One. 2026 Feb 23;21(2):e0343401. doi: 10.1371/journal.pone.0343401 (PMC12928430; doi:10.1371/journal.pone.0343401)

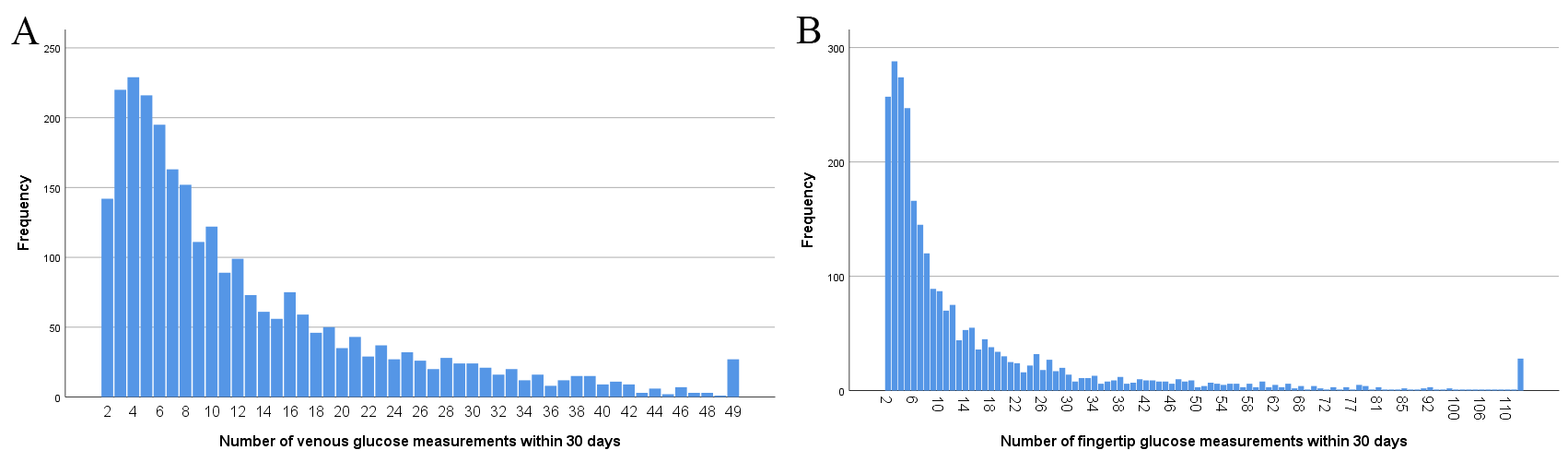

Supplement: S1 Fig — Number of fingertip glucose measurements within 30 day (B). (TIF) [file pone.0343401.s010.tif]

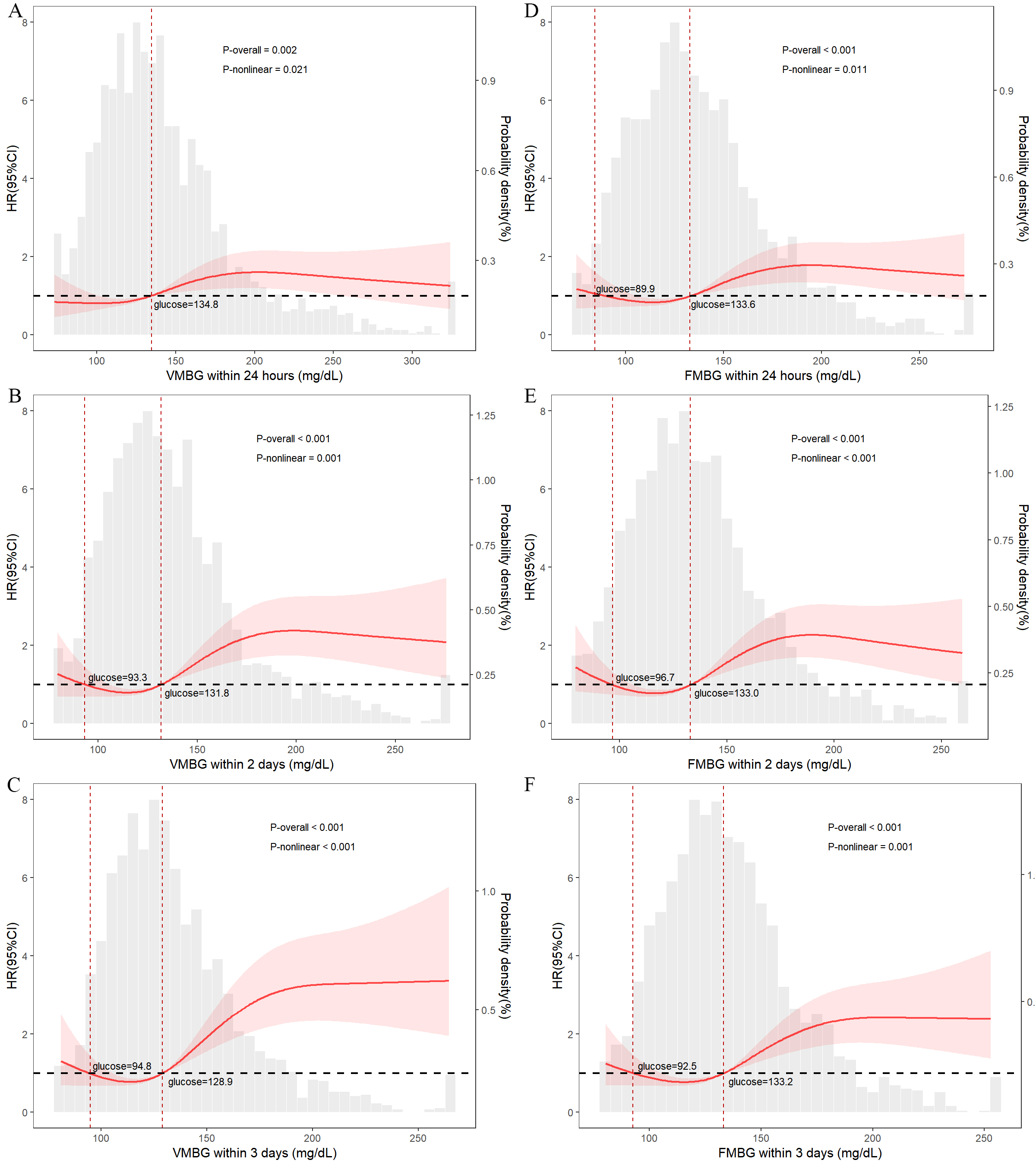

Supplement: S2 Fig — VMBG: mean blood glucose of venous. FMBG: mean blood glucose of fingertip. RCS: restricted cubic splines. (TIF) [file pone.0343401.s011.tif]

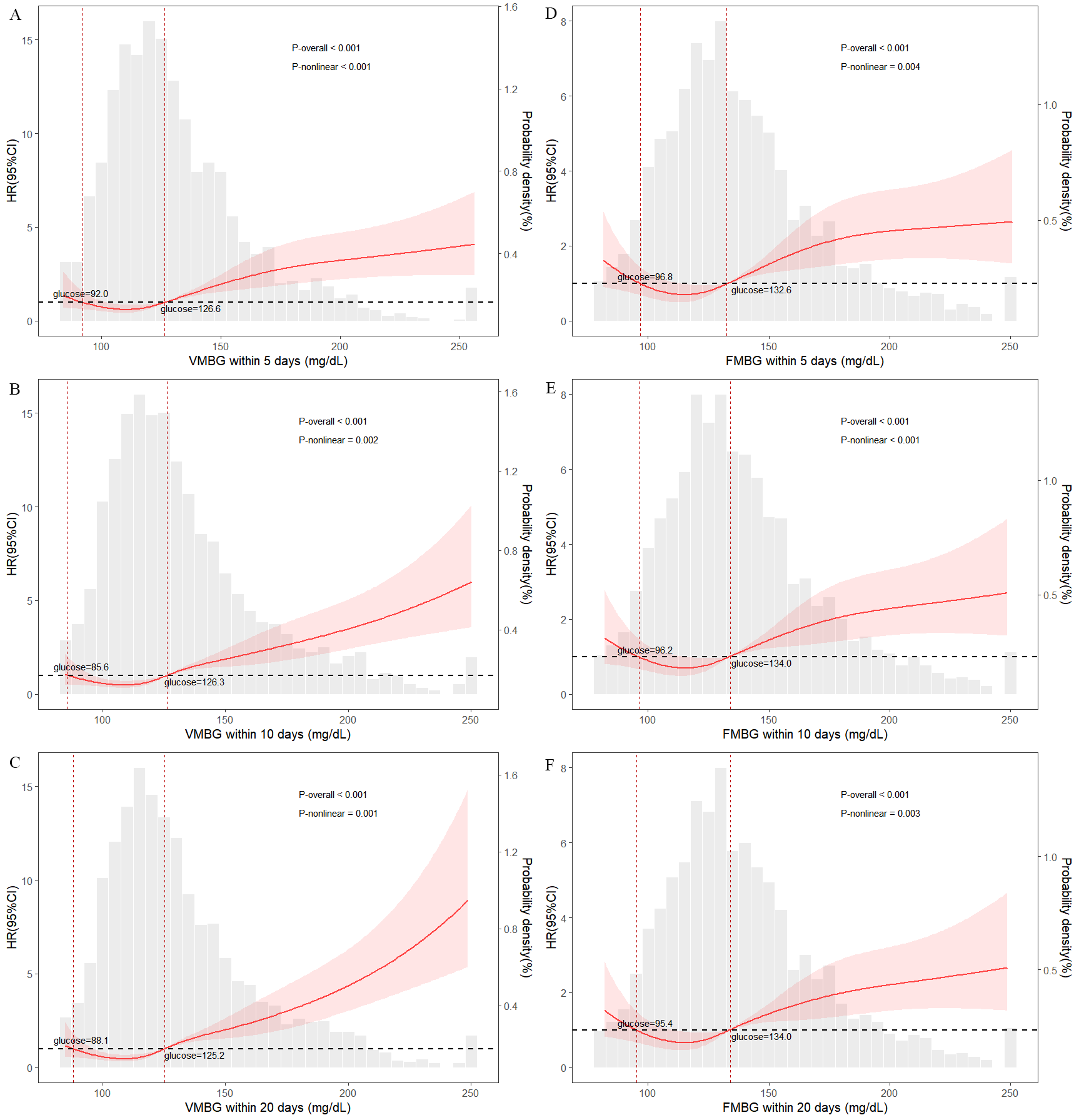

Supplement: S3 Fig — VMBG: mean blood glucose of venous. FMBG: mean blood glucose of fingertip. RCS: restricted cubic splines. (TIF) [file pone.0343401.s012.tif]

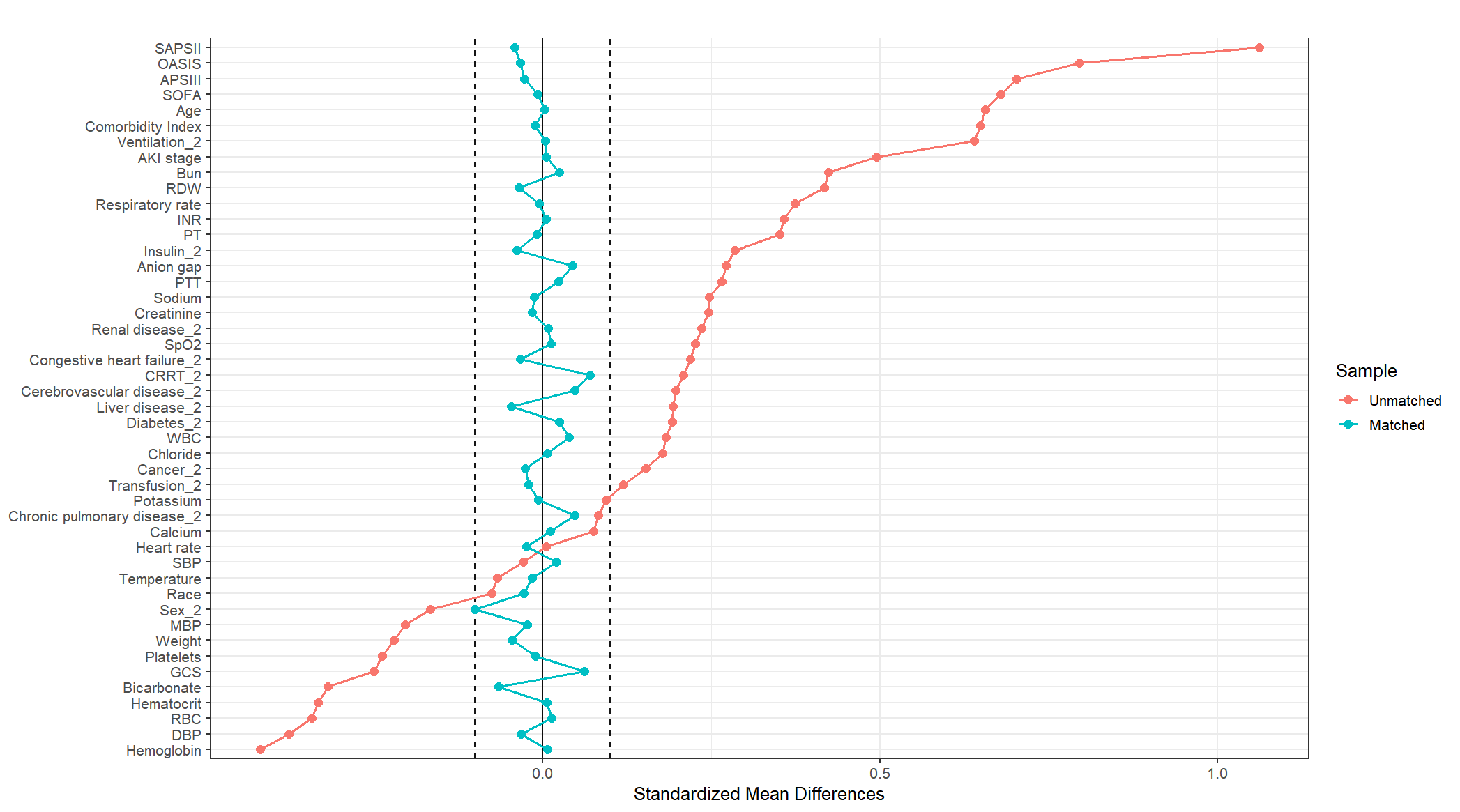

Supplement: S4 Fig — SMDs: standardized mean differences. (TIFF) [file pone.0343401.s013.tiff]
