# Supplementary material for: Differential analysis of mean blood glucose levels from venous and fingertip in predicting 30-day mortality among ICU patients with severe trauma: A retrospective study utilizing the MIMIC-IV database
Source: PLoS One. 2026 Feb 23;21(2):e0343401. doi: 10.1371/journal.pone.0343401 (PMC12928430; doi:10.1371/journal.pone.0343401)
Supplement: Attachment — Submitted filename: Response to Reviewers.docx [file pone.0343401.s016.docx]

Dear editor and reviewers of PLOS ONE:

Our reference: PONE-D-25-23041

Title: Differential analysis of mean blood glucose levels from venous and fingertip in predicting 30-day mortality among ICU patients with severe trauma: A retrospective study utilizing the MIMIC-IV database

By: Yun Liu et al

Thank you very much for your letter and for the editors’ and reviewers’ comments concerning our manuscript entitled "Differential analysis of mean blood glucose levels from venous and fingertip in predicting 30-day mortality among ICU patients with severe trauma: A retrospective study utilizing the MIMIC-IV database" (ID: PONE-D-25-23041). These comments are of great reference value to the revision and improvement of our paper and have important guiding significance to our researches. We have studied comments carefully and have made correction. We hope that the revision is acceptable and look forward to hearing from you soon. Revised portion are marked in color in the paper. The main corrections in the paper and the responds to the journal requirements and reviewer’s comments are as flowing:

**Journal requirements:**

1. Please ensure that your manuscript meets PLOS ONE's style requirements, including those for file naming. The PLOS ONE style templates can be found at

https://journals.plos.org/plosone/s/file?id=wjVg/PLOSOne_formatting_sample_main_body.pdf and <https://journals.plos.org/plosone/s/file?id=ba62/PLOSOne_formatting_sample_title_authors_affiliations.pdf>

We confirm that the manuscript complies with the style requirements of PLOS ONE, including the specified file naming conventions.

2. Thank you for stating the following financial disclosure:

 [The project was supported by the 2024 Excellence Program for Young and Middle-aged Talents at Suzhou Ninth People's Hospital.].

Please state what role the funders took in the study.  If the funders had no role, please state: "The funders had no role in study design, data collection and analysis, decision to publish, or preparation of the manuscript."

If this statement is not correct you must amend it as needed.

Please respond by return e-mail so that we can amend your financial disclosure and competing interests on your behalf.

Our research was supported by the 2024 Excellence Program for Young and Middle-aged Talents at Suzhou Ninth People's Hospital. Award Number: Document No. [2024] 23 of Suzhou Ninth People's Hospital Party Committee. We confirm that the funders had no role in study design, data collection and analysis, decision to publish, or preparation of the manuscript.

3. PLOS requires an ORCID iD for the corresponding author in Editorial Manager on papers submitted after December 6th, 2016. Please ensure that you have an ORCID iD and that it is validated in Editorial Manager. To do this, go to ‘Update my Information’ (in the upper left-hand corner of the main menu), and click on the Fetch/Validate link next to the ORCID field. This will take you to the ORCID site and allow you to create a new iD or authenticate a pre-existing iD in Editorial Manager.

The corresponding author had registered an ORCID iD and successfully updated it in the Editorial Manager of PLOS. The ORCID iD is: https://orcid.org/0009-0005-8521-8723.

4. If the reviewer comments include a recommendation to cite specific previously published works, please review and evaluate these publications to determine whether they are relevant and should be cited. There is no requirement to cite these works unless the editor has indicated otherwise.

The reviewer comments did not indicate the need to cite of specific content from previously published works.

5. Please review your reference list to ensure that it is complete and correct. If you have cited papers that have been retracted, please include the rationale for doing so in the manuscript text, or remove these references and replace them with relevant current references. Any changes to the reference list should be mentioned in the rebuttal letter that accompanies your revised manuscript. If you need to cite a retracted article, indicate the article’s retracted status in the References list and also include a citation and full reference for the retraction notice.

We have conducted a thorough review of the reference list. We can confirm that all references are complete and correct, and to the best of our knowledge, no retracted literature has been cited.

**Reviewers' comments:**

Reviewer #1: The chosen topic is highly relevant in both the ICU and trauma settings, as the research team clearly demonstrated in the introduction. The work is well described, with clear definitions of some concepts.

We would like to express our sincere gratitude to Reviewer #1 for their generous and positive assessment of our work. We are greatly encouraged by their comments that the chosen topic is highly relevant and that the work is well-described with clear definitions. Such acknowledgement is very motivating for our team.

Reviewer #2: Dear authors, in the conclusion of your study you emphasized the limits of this study and I fully agree with what was stated. Please clarify the glycemic profiling and the comparative time of sampling, I cannot establish from your data whether these are average values or the highest values, nor do I have insight into the timeline of sampling.

During the revision process, we incorporated the original data files for venous and fingertip glucose measurements, specifically "raw data of venous glucose within 30 days.csv" and "raw data of fingertip glucose within 30 days.csv." These datasets included information on 2,699 trauma patients, including patient IDs, ICU admission times, all recorded venous glucose values within 30 days, fingertip glucose values within 30 days, and corresponding glucose measurement timestamps. The data were organized sequentially by patient ID and measurement timestamp, enabling a clear and systematic review of each glucose value and its associated time point. As illustrated in Figures R1 and R2, fingertip glucose measurements exhibited a more dispersed distribution across daily intervals, whereas venous glucose measurements were predominantly clustered between 0:00 and 7:00. Further analysis revealed that there was no significant difference in the number of venous glucose measurements between non-survivors and survivors groups within 30 days; however, the number of fingertip glucose measurements in the non-survivor group was higher compared to the survivor group, and both venous and fingertip glucose monitoring frequencies were elevated in the non-survivor group (Table R1). This pattern indicated that glucose monitoring frequency might be determined by clinical necessity. After adjusting for glucose measurement frequency using Model 3, the associations between VMBG and FMBG with 30-day mortality remained statistically significant. Specifically, each 1-unit increase in VMBG was associated with a 1.019-fold increase in mortality risk (HR = 1.019, 95% CI: 1.015–1.022), and each 1-unit increase in FMBG corresponded to a 1.009-fold increase in risk (HR = 1.009, 95% CI: 1.004–1.012) (Tables R2 and R3).

Due to the inherent limitations of retrospective studies, blood glucose measurements were characterized by non-standardized timing and variable resolution. To mitigate potential bias arising from inconsistent measurement protocols, we conducted additional analyses to evaluate the predictive performance of mean blood glucose levels across different time intervals—specifically, within 24 hours, 5 days, 10 days, and 20 days—on 30-day mortality. These analyses included both intergroup and intragroup comparisons. The results were presented in the section titled "The associations between VMBG, FMBG, and 30-day mortality across various time intervals," along with Fig. 4 and Supplementary Table 4.

Furthermore, the VMBG reported in the manuscript represents the mean of all venous blood glucose measurements for each patient over a 30-day period, while the FMBG refer to the mean of all fingertip blood glucose measurements collected during the same period. This clarification had been incorporated into the Patients section under "Methods" to enhance transparency and consistency.

**Fig R1** The time distribution of venous blood glucose measurements within 30 days

All blood glucose measurement times were categorized and tallied by hour from 0 to 23. The horizontal axis indicates the time points of daily fingertip blood glucose measurements, while the vertical axis represents the total number of measurements recorded at each corresponding time point.

**Fig R2** The time distribution of fingertip blood glucose measurements within 30 days

All blood glucose measurement times were categorized and tallied by hour from 0 to 23. The horizontal axis indicates the time points of daily fingertip blood glucose measurements, while the vertical axis represents the total number of measurements recorded at each corresponding time point.

**Table R1** The number and frequency of glucose measurements within 30 days

| **Variables** | **Overall** | **30-day survial** | **30-day mortality** | **p** |
| --- | --- | --- | --- | --- |
| **N** | **2699** | **2361** | **338** |  |
| number of venous glucose measurements | 9[5, 17] | 9[5, 17] | 9[6, 16] | 0.551 |
| number of fingertip glucose measurements | 7[4, 17] | 7[4, 16] | 12[6, 25] | <0.001 |
| frequency of venous glucose measurements | 1.04[0.81, 1.28] | 1.02[0.80, 1.22] | 1.38[1.01, 1.96] | <0.001 |
| frequency of fingertip glucose measurements | 0.95[0.54, 1.73] | 0.88[0.51, 1.58] | 2.09[0.97, 2.97] | <0.001 |

frequency of venous glucose measurements: number of venous glucose measurements/ length of hospital stay. frequency of fingertip glucose measurements: number of fingertip glucose measurements/ length of hospital stay. If the length of hospital stay was greater than 30 days, it would be recorded as 30 days.

**Table R2** Cox regression analysis of VMBG and 30-day mortality after adjusting the frequency of venous glucose measurements

| **VMBG** | **＜88.1mg/dl** | **88.1-125.4mg/dl** | **≥125.4mg/dl** | **Continuous** |
| --- | --- | --- | --- | --- |
| HR(95%CI) | 2.450(1.095,5.481) | 1(reference) | 3.065(2.266,4.146) | 1.019(1.015,1.022) |
| P | 0.029 | - | ＜0.001 | ＜0.001 |

adjusted for sex, age, race, comorbidity index, cerebrovascular disease, liver disease, chronic pulmonary disease, diabetes, congestive heart failure, cancer, renal disease, CRRT, ventilation, insulin, transfusion, SOFA, GCS, AKI stage, SAPSⅡ, APSⅢ, OASIS, frequency of venous glucose measurements.

**Table R3** Cox regression analysis of FMBG and 30-day mortality after adjusting the frequency of fingertip glucose measurements

| **FMBG** | **＜95.4mg/dl** | **95.4-134.0mg/dl** | **≥134.0mg/dl** | **Continuous** |
| --- | --- | --- | --- | --- |
| HR(95%CI) | 1.948(1.161,3.269) | 1(reference) | 1.679(1.161,3.269) | 1.008(1.004,1.012) |
| P | 0.012 | - | ＜0.001 | ＜0.001 |

adjusted for sex, age, race, comorbidity index, cerebrovascular disease, liver disease, chronic pulmonary disease, diabetes, congestive heart failure, cancer, renal disease, CRRT, ventilation, insulin, transfusion, SOFA, GCS, AKI stage, SAPSⅡ, APSⅢ, OASIS, frequency of fingertip glucose measurements.

Once again, thank you very much for your comments and suggestions.

Yours sincerely,

Yun Liu
2025-11-04
