# Supplementary material for: Differential analysis of mean blood glucose levels from venous and fingertip in predicting 30-day mortality among ICU patients with severe trauma: A retrospective study utilizing the MIMIC-IV database
Source: PLoS One. 2026 Feb 23;21(2):e0343401. doi: 10.1371/journal.pone.0343401 (PMC12928430; doi:10.1371/journal.pone.0343401)
Supplement: Attachment — Submitted filename: PONE-D-25-23041R1.docx [file pone.0343401.s017.docx]

# My Report:

I commend the authors for addressing an important and clinically meaningful question regarding differential prognostic value of venous versus fingertip mean blood glucose in critically ill trauma patients. The large sample size, comprehensive statistical framework, and multiple sensitivity analyses are notable strengths. Nevertheless, several methodological and reporting issues in the Materials and Methods and Results sections should be addressed to improve transparency, reproducibility, and interpretability.

**1.** The definition of VMBG and FMBG as mean glucose values over the same 30-day period used for the primary outcome introduces potential temporal overlap between exposure and outcome. This raises concerns regarding reverse causation and immortal time bias. Additional clarification or justification is needed, and stronger emphasis on early-interval analyses is recommended.

**2.** The lack of standardized glucose measurement frequency may bias mean glucose estimates, particularly since sicker patients are more likely to undergo frequent testing. This issue should be more explicitly acknowledged and discussed as a methodological limitation.

**3.** Although glycemic variability is defined and reported, its role in the analytical framework is unclear, as it is not incorporated into the primary multivariable models. A clearer rationale for its inclusion or exclusion is warranted.

**4.** The choice of a relatively wide caliper (0.4) requires justification. While preserving sample size is important, readers would benefit from an explanation of the balance–precision trade-off underlying this decision.

**5.** Although statistically significant differences between VMBG and FMBG are observed, both AUCs indicate only moderate discrimination. A brief comment on clinical relevance versus statistical significance would strengthen the results interpretation.

**6.** The identification of J-shaped associations using RCS is a major strength. However, the derived glucose thresholds are data-driven and should be reported with caution, emphasizing their exploratory rather than prescriptive nature.

**7.** The observed interactions with age, race, and diabetes status are interesting but should be interpreted cautiously due to multiple comparisons. These findings should be clearly labeled as exploratory.

**8.** The increasing predictive performance with longer glucose observation windows is expected but further highlights temporal overlap concerns. These results are best framed as predictive rather than causal.
