# Supplementary material for: Differential analysis of mean blood glucose levels from venous and fingertip in predicting 30-day mortality among ICU patients with severe trauma: A retrospective study utilizing the MIMIC-IV database
Source: PLoS One. 2026 Feb 23;21(2):e0343401. doi: 10.1371/journal.pone.0343401 (PMC12928430; doi:10.1371/journal.pone.0343401)
Supplement: Attachment — Submitted filename: Response to Reviewers R1.docx [file pone.0343401.s018.docx]

Dear editor and reviewers of PLOS ONE:

Our reference: PONE-D-25-23041R1

Title: Differential analysis of mean blood glucose levels from venous and fingertip in predicting 30-day mortality among ICU patients with severe trauma: A retrospective study utilizing the MIMIC-IV database

By: Yun Liu et al

Thank you very much for your letter and for the editors’ and reviewers’ comments concerning our manuscript entitled "Differential analysis of mean blood glucose levels from venous and fingertip in predicting 30-day mortality among ICU patients with severe trauma: A retrospective study utilizing the MIMIC-IV database" (ID: PONE-D-25-23041R1). These comments are of great reference value to the revision and improvement of our paper and have important guiding significance to our researches. We have studied comments carefully and have made correction. We hope that the revision is acceptable and look forward to hearing from you soon. Revised portion are marked in color in the paper. The main corrections in the paper and the responds to the journal requirements and reviewer’s comments are as flowing:

**Reviewers' comments:**

Reviewer #3: 

1. The definition of VMBG and FMBG as mean glucose values over the same 30-day period used for the primary outcome introduces potential temporal overlap between exposure and outcome. This raises concerns regarding reverse causation and immortal time bias. Additional clarification or justification is needed, and stronger emphasis on early-interval analyses is recommended.

We sincerely thank the reviewer for raising the crucial methodological point regarding temporal overlap, reverse causation and immortal time bias. In the revised version, we had presented a comprehensive description of the correlation analysis between early-interval glucose exposure and prognosis. The main research had been modified to: To compare the predictive efficacy of mean blood glucose of venous (VMBG) and mean blood glucose of fingertip (FMBG) in assessing the 30-days mortality risk of ICU trauma patients, and to systematically evaluate the prognostic value of early - stage (24-hour, 2-day, 3-day) and long - term (30-day) monitoring intervals. Specifically, we had performed new multivariable analyses demonstrating that the mean blood glucose levels during the initial 24-hour, 2-day, and 3-day periods following admission were also significantly associated with 30-day mortality (Table 1).

We had highlighted the research findings from the early-interval glucose monitoring windows (24-hour, 2-day, and 3-day) because these results provide greater methodological advantages in avoiding time overlap. We presented the information in the section titled "The associations between VMBG, FMBG of various time intervals and 30-day mortality". We also observed that within 24 hours, the VMBG presented only a single risk threshold, whereas the FMBG had already demonstrated typical dual risk thresholds of low and high (Fig 1). This discrepancy was likely attributable to their distinct physiological responses in the acute pathological setting. VMBG primarily reflected the systemic hyperglycemic load driven by a severe stress response (e.g., catecholamine and cortisol surge) [1]. In contrast, FMBG was more susceptible to local interference under conditions of early microcirculatory instability and potentially compromised peripheral perfusion. It had been documented that, compared to arterial levels, FMBG may significantly underestimate blood glucose in patients with shock or on vasopressors [2]. Consequently, the earlier emergence of a low-glucose threshold in FMBG could have served as an early warning signal of peripheral hypoperfusion. With time, the risk models revealed by the two measurement methods converged, both demonstrating a clear ‘J-shaped’ dual-threshold association.

Concurrently, we retained the sensitivity analysis, PSM analysis, and subgroup analysis for VMBG and FMBG of 30-day. Despite a certain degree of temporal overlap, these exploratory analyses, to varying extents, disclosed the correlation between blood glucose and prognosis and maintained a high level of consistency and robustness, thereby offering hypotheses for further research.

2. The lack of standardized glucose measurement frequency may bias mean glucose estimates, particularly since sicker patients are more likely to undergo frequent testing. This issue should be more explicitly acknowledged and discussed as a methodological limitation.

We thank the reviewer for this crucial methodological insight. We fully agree that the lack of standardized glucose measurement frequency is an important limitation of our retrospective study, as sicker patients indeed undergo more frequent testing, which could introduce surveillance bias and potentially overestimate the association between glucose indices and mortality. We have now explicitly acknowledged and discussed this limitation in the revised Discussion/Limitations section. We further note that future prospective studies with standardized measurement protocols are needed to confirm our findings.

3. Although glycemic variability is defined and reported, its role in the analytical framework is unclear, as it is not incorporated into the primary multivariable models. A clearer rationale for its inclusion or exclusion is warranted.

We sincerely thank the reviewer for raising this important point. Our decision not to incorporate glycemic variability (GV) into the primary multivariate models was based on a comprehensive assessment including clinical relevance, statistical metrics, and methodological considerations, as detailed below:

One of the objectives of our study was to directly compare the prognostic value of mean blood glucose from two different sampling sites (venous vs. fingertip). In the exploratory analysis, GV was incorporated into the multivariate model. Although GV also demonstrated statistical significance, it did not significantly alter the HRs and statistical significance of the mean blood glucose variables(Table 2, Table3). Furthermore, as a result of including the variable GV associated with mean blood glucose, the complexity of the model also rose.

We quantified the additional predictive value of GV. As shown in Tables 2 and 3, when comparing Model a2 with Model a3, and Model b2 with Model b3, there were only minimal changes in their Akaike Information Criterion (AIC) and C - index. Additionally, we utilized the survIDINRI package in R to calculate the net reclassification index (NRI) and the integrated discrimination improvement index (IDI) for model comparison. The results indicated that there was no significant improvement in the NRI and IDI of Model a3 relative to Model a2, with values of 0.052 (-0.101 - 0.128) p = 0.252 and 0.003 (-0.001 - 0.010) p = 0.153, respectively. Similarly, there was no significant improvement in the NRI and IDI of Model b3 relative to Model b2, with values of 0.28 (-0.059 - 0.085) p = 0.292 and 0.003 (-0.001 - 0.013) p = 0.106, respectively. Collectively, these indicators suggested that, within the multivariate model of this cohort, neither VGV nor FGV provided substantial additional prognostic information for our primary outcome indicators.

 GV was defined as the ratio of the standard deviation (SD) of blood glucose levels to the mean blood glucose (MBG) concentration, and it served as a proportional covariate. When both GV and MBG were incorporated into the regression model simultaneously, although the VIF values wew both less than 10, a certain mathematical coupling exists. For instance, when interpreting the coefficient of MBG, GV was held at a fixed value, and it was necessary to assume that SD varies strictly in a fixed proportion with MBG. This situation did not align with biological reality. As Valle et al. had expressed concerns, the inclusion of proportional covariates in the regression model complicated model interpretation[3]. Furthermore, the fixed proportion of GV could manifest as either high SD/high MBG or low SD/low MBG. It remained to further study whether these two blood glucose patterns exert different physiological and pathological effects.

4. The choice of a relatively wide caliper (0.4) requires justification. While preserving sample size is important, readers would benefit from an explanation of the balance–precision trade-off underlying this decision.

We utilized the nearest neighbor algorithm for propensity score matching (PSM). Although the commonly recommended caliper width was 0.2[4], to optimize the balance–precision trade-off and to prevent a substantial number of sample losses in the limited overlapping areas within our cohort, we selected a matching width of 0.4. The chosen caliper width of 0.4 achieved an excellent balance of covariates while retaining a sufficient quantity of matches, and all the post - matching standardized mean differences (SMDs) were less than 0.1. This balance was verified through a sensitivity analysis employing a 0.2 caliper width (Fig 2).

In the "Statistical analysis" section of the original manuscript, we have modified it to: To optimize the balance–precision trade-off, propensity score matching (PSM) analysis was conducted using a nearest-neighbor matching algorithm with a caliper width of 0.4 at a 2:1 ratio. And a sensitivity analysis was conducted using caliper width of 0.2.

5. Although statistically significant differences between VMBG and FMBG are observed, both AUCs indicate only moderate discrimination. A brief comment on clinical relevance versus statistical significance would strengthen the results interpretation.

We acknowledged that while the statistically significant difference in AUC between VMBG and FMBG was observed, the absolute values indicated only moderate discriminatory power, meaning neither metric was suitable as a standalone, perfect predictor for individual prognosis. Thus, the significance of our work lay in refining monitoring strategies and building a more nuanced foundation for risk assessment, rather than offering a single diagnostic tool.

6. The identification of J-shaped associations using RCS is a major strength. However, the derived glucose thresholds are data-driven and should be reported with caution, emphasizing their exploratory rather than prescriptive nature.
We thank the reviewer for this important point. We agreed the derived glucose thresholds were data-driven. In our revision, we explicitly reported them with caution. We labeled them as exploratory and hypothesis generating, not prescriptive clinical targets. We emphasized the biological insight from the J-shaped pattern over the specific threshold values. These thresholds require external validation but help advance the pathophysiological understanding of dysglycemia in trauma.

7. The observed interactions with age, race, and diabetes status are interesting but should be interpreted cautiously due to multiple comparisons. These findings should be clearly labeled as exploratory.

We fully agreed that the observed interactions with age, race, and diabetes status should be interpreted with great caution. In the revised manuscript, we had clearly designated these findings as exploratory in both the results section and the discussion section. In the discussion section, we have re - elaborated on the interpretation method, indicating that within these subgroups, the relationship between blood sugar and prognosis may be particularly significant and merits specialized future research, rather than being considered as the final conclusion.

8. The increasing predictive performance with longer glucose observation windows is expected but further highlights temporal overlap concerns. These results are best framed as predictive rather than causal.

We appreciated the reviewer's important point. We agreed that as the observation windows became longer and the glucose information accumulated, the predictive performance increased. Although this was expected, it highlighted the core issue of temporal overlap. In response, we reframed these specific findings in the revised Discussion as demonstrating a predictive association rather than implying causality. We contrasted this with our analysis of early glucose windows, which was designed to better support causal hypotheses. Finally, we stated that any causal inference would require prospective interventional trials.

**Table 1** Multivariate COX regression analysis of VMBG and FMBG at different time intervals

| Time Intervals | VMBG | | FMBG | |
| --- | --- | --- | --- | --- |
|  | HR(95%CI) | P | HR(95%CI) | P |
| within 24 hours (mg/dL) | 1.003(1.001,1.006) | 0.006 | 1.004(1.002,1.007) | 0.002 |
| within 48 hours (mg/dL) | 1.007(1.004,1.010) | ＜0.001 | 1.007(1.003,1.010) | ＜0.001 |
| within 72 hours (mg/dL) | 1.010(1.007,1.013) | ＜0.001 | 1.009(1.005,1.012) | ＜0.001 |
| within 5 days (mg/dL) | 1.013(1.009,1.016) | ＜0.001 | 1.009(1.005,1.013) | ＜0.001 |
| within 10 days (mg/dL) | 1.016(1.012,1.020) | ＜0.001 | 1.009(1.006,1.013) | ＜0.001 |
| within 20 days (mg/dL) | 1.019(1.015,1.022) | ＜0.001 | 1.009(1.005,1.013) | ＜0.001 |
| within 30 days (mg/dL) | 1.019(1.016,1.023) | ＜0.001 | 1.009(1.006,1.013) | ＜0.001 |

VMBG: mean blood glucose of venous. FMBG: mean blood glucose of fingertip. Adjusted for sex, age, race, comorbidity index, cerebrovascular disease, liver disease, chronic pulmonary disease, diabetes, congestive heart failure, cancer, renal disease, CRRT, ventilation, insulin, transfusion, SOFA, GCS, AKI stage, SAPSⅡ, APSⅢ, OASIS

**Table 2** Exploratory analysis of VMBG and VGV in the multivariate model

| Model | VMBG | | | VGV | | | AIC | C-index |
| --- | --- | --- | --- | --- | --- | --- | --- | --- |
|  | HR(95% CI) | P | VIF | HR(95% CI) | P | VIF |  |  |
| Model a1 | 1.018(1.707,2.112) | <0.001 | 1.273 | 0.996(0.857,1.048) | 0.299 | 1.273 | 5161.582 | 0.696 |
| Model a2 | 1.019(1.016,1.023) | <0.001 | 1.642 | - | - | - | 4780.341 | 0.833 |
| Model a3 | 1.022(1.018,1.026) | <0.001 | 2.043 | 0.988(0.978,0.997) | 0.011 | 1.427 | 4775.493 | 0.834 |

Model a1: VMBG adjusted for VGV. Model a2: VMBG adjusted for Covariates. Model a3: VMBG adjusted for VGV and Covariates. The Covariates included sex, age, race, comorbidity index, cerebrovascular disease, liver disease, chronic pulmonary disease, diabetes, congestive heart failure, cancer, renal disease, CRRT, ventilation, insulin, transfusion, SOFA, GCS, AKI stage, SAPSⅡ, APSⅢ, OASIS. VMBG: mean blood glucose of venous within 30 days. VGV: glycemic variability of venous within 30 days. Akaike Information Criterion (AIC). Variance Inflation Factor (VIF).

**Table 3** Exploratory analysis of FMBG and FGV in the multivariate model

| Model | FMBG | | | FGV | | | AIC | C-index |
| --- | --- | --- | --- | --- | --- | --- | --- | --- |
|  | HR(95% CI) | P | VIF | HR(95% CI) | P | VIF |  |  |
| Model b1 | 1.009(1.006,1.011) | <0.001 | 1.132 | 1.026(1.015,1.037) | <0.001 | 1.132 | 5224.042 | 0.648 |
| Model b2 | 1.009(1.006,1.013) | <0.001 | 1.637 | - | - | - | 4860.390 | 0.816 |
| Model b3 | 1.009(1.002,1.013) | <0.001 | 1.658 | 1.014(1.002,1.028) | 0.026 | 1.383 | 4857.521 | 0.816 |

Model b1: FMBG adjusted for FGV. Model b2: FMBG adjusted for Covariates. Model b3: FMBG adjusted for FGV and Covariates. The Covariates included sex, age, race, comorbidity index, cerebrovascular disease, liver disease, chronic pulmonary disease, diabetes, congestive heart failure, cancer, renal disease, CRRT, ventilation, insulin, transfusion, SOFA, GCS, AKI stage, SAPSⅡ, APSⅢ, OASIS. FMBG: mean blood glucose of fingertip within 30 days. FGV : glycemic variability of fingertip within 30 days. Akaike Information Criterion (AIC). Variance Inflation Factor (VIF).

**Fig 1 RCS analysis of the correlation between VMBG, FMBG of various early-time intervals and 30-day mortality**

VMBG: mean blood glucose of venous. FMBG: mean blood glucose of fingertip. RCS: restricted cubic splines.

**Fig 2. The AUCs (A) and multivariate adjusted COX regression analysis (B) of VMBG and FMBG in different time intervals post-ICU admission for predicting 30-day all-cause mortality**. Adjusted for sex, age, race, comorbidity index, cerebrovascular disease, liver disease, chronic pulmonary disease, diabetes, congestive heart failure, cancer, renal disease, CRRT, ventilation, insulin, transfusion, SOFA, GCS, AKI stage, SAPSⅡ, APSⅢ, OASIS. AUC: area under curve.

**Fig 2** SMDs of variables prior to and following the matching process with a caliper width of 0.2. SMDs: standardized mean differences.

**References**

[1] El-Menyar A, Asim M, Mir F, Hakim S, Kanbar A, Siddiqui T, et al. Patterns and Effects of Admission Hyperglycemia and Inflammatory Response in Trauma Patients: A Prospective Clinical Study. World J Surg. 2021;45(9):2670-2681. doi: 10.1007/s00268-021-06190-5. PMID: 34117510.
[2] Pulzi Júnior SA, Assunção MS, Mazza BF, Fernandes Hda S, Jackiu M, Freitas FG, et al. Accuracy of different methods for blood glucose measurement in critically ill patients. Sao Paulo Med J. 2009;127(5):259-65. doi: 10.1590/s1516-31802009000500003. PMID: 20169273.
[3] Valle D, Mintz J, Brack IV. Estimation and interpretation problems and solutions when using proportion covariates in linear regression models. Ecology. 2024;105(4):e4256. doi: 10.1002/ecy.4256. PMID: 38361276.
[4] Austin PC. Optimal caliper widths for propensity-score matching when estimating differences in means and differences in proportions in observational studies. Pharm Stat. 2011;10(2):150-61. doi: 10.1002/pst.433. PMID: 20925139.

Once again, thank you very much for your comments and suggestions.

Yours sincerely,

Yun Liu
2026-01-20
