# Supplementary material for: Differential analysis of mean blood glucose levels from venous and fingertip in predicting 30-day mortality among ICU patients with severe trauma: A retrospective study utilizing the MIMIC-IV database
Source: PLoS One. 2026 Feb 23;21(2):e0343401. doi: 10.1371/journal.pone.0343401 (PMC12928430; doi:10.1371/journal.pone.0343401)
Supplement: Attachment — Submitted filename: My report, PONE-D-25-23041_R2.docx [file pone.0343401.s019.docx]

# My Report:

This manuscript leverages the large, contemporary, and high-quality MIMIC-IV database and applies a comprehensive suite of modern statistical techniques, including time-to-event modeling, restricted cubic splines, propensity score matching, and multiple sensitivity analyses. The systematic comparison between venous and fingertip mean blood glucose as prognostic indicators in ICU trauma patients is both novel and clinically relevant. Overall, the study is methodologically up-to-date, well-powered, and addresses an important gap in the literature regarding modality-specific glycemic assessment in critically injured patients.

## Comments

1. The inclusion criterion requiring at least two glucose measurements within 30 days may introduce substantial heterogeneity in the precision of mean blood glucose estimation. Reporting the distribution of glucose measurement frequency per patient would help readers assess the robustness of MBG estimates.

2. Glycemic variability is defined as SD divided by the mean glucose, which introduces intrinsic mathematical coupling with MBG. Although GV was not included in the primary multivariable models, this dependency should be explicitly acknowledged as a methodological limitation.

3. The manuscript does not clarify the clinical conditions under which venous and fingertip glucose measurements were obtained (e.g., shock states, vasopressor use, hypothermia). These factors may systematically bias fingertip glucose values and warrant explicit discussion in the Methods section.

4. Multiple severity-of-illness scores (SOFA, SAPS II, APS III, and OASIS) are simultaneously included in the adjusted models. Given their conceptual overlap, an assessment of collinearity or a justification for retaining all scores concurrently would improve model transparency.

6. Extreme value handling using 1% tail trimming is mentioned, but the specific variables affected and the impact of this decision on effect estimates are not reported. A brief sensitivity assessment would enhance reproducibility.

7. Although early time-window analyses were conducted to mitigate immortal time bias, the application of formal landmark analysis or time-dependent Cox models could further strengthen causal interpretability.

8. While the difference in AUC between VMBG and FMBG is statistically significant, the absolute values indicate only moderate discrimination. A clearer discussion of clinical relevance versus statistical significance would improve result interpretation.

9. The progressive increase in hazard ratios with longer glucose observation windows is expected and likely reflects cumulative prognostic information. These findings should be clearly distinguished as predictive rather than causal.

10. Subgroup analyses by age, race, and diabetes status are intriguing but involve multiple comparisons. These results should be explicitly labeled as hypothesis-generating.

11. Given the known limitations of fingertip glucose measurements under conditions of impaired peripheral perfusion, stratified analyses among patients receiving vasopressors or with severe AKI could further contextualize the findings.

12. The interpretation of ROC analyses alongside survival models would be strengthened by reporting model calibration metrics, such as calibration plots or Brier scores.
