# Supplementary material for: Differential analysis of mean blood glucose levels from venous and fingertip in predicting 30-day mortality among ICU patients with severe trauma: A retrospective study utilizing the MIMIC-IV database
Source: PLoS One. 2026 Feb 23;21(2):e0343401. doi: 10.1371/journal.pone.0343401 (PMC12928430; doi:10.1371/journal.pone.0343401)
Supplement: Attachment — Submitted filename: Response to Reviewers R2.docx [file pone.0343401.s020.docx]

Dear editor and reviewers of PLOS ONE:

Our reference: PONE-D-25-23041R2

Title: Differential analysis of mean blood glucose levels from venous and fingertip in predicting 30-day mortality among ICU patients with severe trauma: A retrospective study utilizing the MIMIC-IV database

By: Yun Liu et al

Thank you very much for your letter and for the editors’ and reviewers’ comments concerning our manuscript entitled "Differential analysis of mean blood glucose levels from venous and fingertip in predicting 30-day mortality among ICU patients with severe trauma: A retrospective study utilizing the MIMIC-IV database" (ID: PONE-D-25-23041R2). We have studied comments carefully and have made correction. We hope that the revision is acceptable and look forward to hearing from you soon. Revised portion are marked in color in the paper. The main corrections in the paper and the responds to the journal requirements and reviewer’s comments are as flowing:

**Comments:**

1.The inclusion criterion requiring at least two glucose measurements within 30 days may introduce substantial heterogeneity in the precision of mean blood glucose estimation. Reporting the distribution of glucose measurement frequency per patient would help readers assess the robustness of MBG estimates.

All patients underwent a minimum of two venous blood glucose measurements and two fingertip blood glucose measurements during the 30-day observation period. The quantity of venous blood glucose measurements was 9[5, 17], and 2337 cases (86.6%) had four or more measurements. The quantity of fingertip blood glucose measurements was 7[4, 17], and 2154 patients (79.8%) had four or more measurements. The distribution of glucose measurement frequencies for all patients was presented in Figure 1. We had made revisions in the **"Results/Baseline characteristics" section, lines 170-174** of the manuscript.

2.Glycemic variability is defined as SD divided by the mean glucose, which introduces intrinsic mathematical coupling with MBG. Although GV was not included in the primary multivariable models, this dependency should be explicitly acknowledged as a methodological limitation.

Within the **"Methods/Statistical Analysis" section, lines 149-151**, it was explicitly stated that there exists an intrinsic mathematical correlation between MBG and GV. Owing to this methodological constraint, it was not incorporated into the main multivariate model to prevent the emergence of interpretational challenges.

3.The manuscript does not clarify the clinical conditions under which venous and fingertip glucose measurements were obtained (e.g., shock states, vasopressor use, hypothermia). These factors may systematically bias fingertip glucose values and warrant explicit discussion in the Methods section.

We express our sincere gratitude to the reviewers for highlighting this critical point. We fully concur that the clinical status during measurement, such as shock states, vasopressor use, hypothermia, was one of the factor that systematically impacts the accuracy of fingertip blood glucose measurement, and it was necessary to conduct a clear discussion. To address this, we had made the following augmentations in the revised version: In the **"Methods/Patients" section, lines 125-127**, we had included a clarification indicating that the blood glucose data in this study was sourced from routine clinical records, and the measurement conditions were non - standardized. We also recognized the potential limitations of fingertip glucose in situations like shock, the utilization of vasoactive drugs and hypothermia. In the **"Discussion" section, lines 484-492**, we had specifically inserted a paragraph to thoroughly explore the potential bias effect of the peripheral perfusion status on the fingertip glucose value and expound on it as one of the mechanisms that may account for the disparity between fingertip and venous glucose results. Meanwhile, we emphasized the significance of taking the patient's circulatory status into account during clinical interpretation.

4.Multiple severity-of-illness scores (SOFA, SAPS II, APS III, and OASIS) are simultaneously included in the adjusted models. Given their conceptual overlap, an assessment of collinearity or a justification for retaining all scores concurrently would improve model transparency.

To assess collinearity, the variance inflation factors (VIF) for the covariates incorporated in the multivariate model (including SOFA, SAPS II, APS III, and OASIS) were calculated (Table 1). The VIF values for all covariates were below 5, suggesting that no severe multicollinearity was present. This diagnostic outcome had been elaborated on in the **"Statistical Methods" section, lines 163-165**.

6.Extreme value handling using 1% tail trimming is mentioned, but the specific variables affected and the impact of this decision on effect estimates are not reported. A brief sensitivity assessment would enhance reproducibility.

In the "Statistical Methods" section, it had been clearly stated that for continuous variables, a 1% tail trimming was applied to mitigate the impact of extreme values. In response to this concern, an additional sensitivity analysis was conducted: the main model was run using the original data without 1% tail trimming. The results were largely consistent with those of the main analysis, indicating that the conclusions drawn were not sensitive to the treatment of extreme values (Table2 and Table3). The results of this analysis had been included in the **"Results/Sensitivity analysis" section, lines 339-342**, and the table was presented as supplementary material.

7.Although early time-window analyses were conducted to mitigate immortal time bias, the application of formal landmark analysis or time-dependent Cox models could further strengthen causal interpretability.

To conduct a more rigorous assessment of the association between early blood glucose levels and long-term mortality risk and effectively mitigate the immortal time bias, this study further performed a Landmark analysis. The Landmark time point was designated as the third day subsequent to the patient's admission. A new analysis cohort was established which encompassed all patients who survived until the third day and had not been discharged (n = 2516, accounting for 93.2% of the original cohort). In this cohort, VMBG and FMBG for each patient were calculated within the initial 3 days following admission to the ICU. The results demonstrated that the VMBG and FMBG of the deceased group were significantly higher than those of the survivors (Table 4). Subsequently, these two indicators were respectively utilized as exposure variables. Multivariate Cox proportional hazards regression model was applied to evaluate their association with the mortality risk from the Landmark time point (the third day after admission) to the end of the 30th day. The analysis revealed that both VMBG and FMBG within 3 days were significantly positively correlated with the 30-day mortality risk: the adjusted hazard ratio (HR) for VMBG was 1.006 (95% CI: 1.003–1.010), and the adjusted HR for FMBG was 1.005 (95% CI: 1.001–1.009) (Table 5). When the sensitivity analysis was conducted at the landmark time point designated as the second day after the patient's admission, the results were also encouraging. Since patients with a hospital stay of less than 24 hours had been excluded, the VMBG and FMBG within 24 hours had already been analyzed as the Landmark analysis.The above modifications were located in the **"Results" section, lines 312-330** and **"Discussion" section, lines 478-484** of the manuscript.

8.While the difference in AUC between VMBG and FMBG is statistically significant, the absolute values indicate only moderate discrimination. A clearer discussion of clinical relevance versus statistical significance would improve result interpretation.

We thank the reviewer for raising this point regarding the clinical interpretation of the AUC values. We addressed this same comment in detail during the previous revision round. As noted in our prior response and in the revised manuscript, we had expanded the interpretation to clearly distinguish statistical significance from clinical utility. In accordance with the current suggestions, we have adjusted the positions of the paragraphs and made further revisions to the wording, thereby enabling readers to perceive this difference more clearly. **("Discussion" section, lines 498-505)**

9.The progressive increase in hazard ratios with longer glucose observation windows is expected and likely reflects cumulative prognostic information. These findings should be clearly distinguished as predictive rather than causal.

We thank the reviewers for their clarification. We had clearly distinguished this in the **"Discussion" section, lines 494-497.** As the observation time window was extended, the predictive efficacy improved, which was likely to reflect the increased prognostic information contained in "cumulative glycemic load". We explicitly stated that these analytical results should be interpreted as a strong predictive association, and the causal explanation requires prospective intervention studies to verify.

10.Subgroup analyses by age, race, and diabetes status are intriguing but involve multiple comparisons. These results should be explicitly labeled as hypothesis-generating.

In the revised version, we had explicitly marked the subgroup analysis sections associated with age, race, and diabetes status as "exploratory subgroup analysis" **("Results" section, lines 374-386)**. Additionally, in the **"Discussion" section, lines 534-536**, we had emphasized that these results were hypothesis - generating and had not undergone correction for multiple comparisons. Consequently, they required independent verification in future studies.

11. Given the known limitations of fingertip glucose measurements under conditions of impaired peripheral perfusion, stratified analyses among patients receiving vasopressors or with severe AKI could further contextualize the findings.

Owing to the limitations of the retrospective study grounded in the database, we chose the AKI indicator to perform a stratified analysis of fingertip blood glucose measurement. In the Figure 2, we incorporated subgroup and interaction analyses of VMBG, FMBG, and AKI. Venous blood glucose was less influenced by local factors and can more precisely reflect the actual blood glucose level within the body. The subgroup analysis demonstrated that irrespective of whether the patient developed AKI, the venous blood glucose level was significantly associated with the risk of death, indicating a severe stress response and metabolic disorders. While FMBG was associated with the 30-day mortality rate in the AKI group, there was no apparent correlation in the non-AKI group. AKI was not merely a renal function impairment but also signified a systemic severe inflammatory response, endothelial damage, and microcirculation disorder[1]. At this juncture, the value of fingertip blood glucose not only represented the blood glucose concentration but also encompassed information regarding poor tissue perfusion and cellular metabolic disorder[2], thereby endowing it with a stronger prognostic signal. This instability also indirectly validated that the value of fingertip blood glucose as a predictor was not as significant as that of venous blood glucose. **("Results" section, line 388 and "Discussion" section, lines 523-533)**

12. The interpretation of ROC analyses alongside survival models would be strengthened by reporting model calibration metrics, such as calibration plots or Brier scores.

We express our sincere gratitude for the constructive suggestion put forward by the reviewer. We documented the calibration metrics of the survival models for the original cohort and the Landmark cohort using the Brier score. This documentation was presented in **Results section, lines 228-229**, as well as in **Supplementary Table 2** and **Supplementary Table 4**. The Brier scores of these models were all below 0.10, suggesting a high level of consistency between the predicted probabilities and the observed outcomes.

**Table 1** The multiple severity-of-illness scores VIF values of the multivariate models of VMBG and FMBG

| **Multiple severity-of-illness scores** | **VMBG model**  **VIF** | **FMBG model**  **VIF** |
| --- | --- | --- |
| SOFA | 2.55 | 2.50 |
| SAPSⅡ | 4.29 | 4.11 |
| APSⅢ | 3.88 | 3.80 |
| OASIS | 2.30 | 2.28 |

**Table 2** The comparison between VMBG and FMBG within 30 days without trimming

| **Variables** | **Overall** | **30-day survial** | **30-day mortality** | **p** |
| --- | --- | --- | --- | --- |
| N | 2699 | 2361 | 338 |  |
| VMBG | 125.14 [110.84, 147.47] | 122.56 [109.50, 142.83] | 146.56 [127.62, 175.77] | <0.001 |
| FMBG | 133.60 [116.89, 156.40] | 131.82 [115.50, 153.83] | 150.66 [129.50, 174.64] | <0.001 |

**Table 3** Multivariate Cox regression analysis of VMBG, FMBG and 30-day mortality without trimming

| **Models** | **HR(95%CI)** | **P** | **Brier** |
| --- | --- | --- | --- |
| VMBG | 1.010(1.001,1.012) | ＜0.001 | 0.084 |
| FMBG | 1.008(1.005,1.011) | ＜0.001 | 0.087 |

Adjusted for sex, age, race, comorbidity index, cerebrovascular disease, liver disease, chronic pulmonary disease, diabetes, congestive heart failure, cancer, renal disease, CRRT, ventilation, insulin, transfusion, SOFA, GCS, AKI stage, SAPSⅡ, APSⅢ, OASIS.

**Table 4** The comparison between VMBG, FMBG and 30-day mortality of the landmark cohorts

| **Variables** | **Overall** | **30-day survial** | **30-day mortality** | **p** |
| --- | --- | --- | --- | --- |
| Landmark of 2 days’ point | |  |  |  |
| N | 2634 | 2332 | 302 |  |
| VMBG | 132.00 [113.33, 155.00] | 130.00 [112.50, 153.00] | 147.06 [124.58, 170.69] | <0.001 |
| FMBG | 133.40 [114.00, 156.11] | 131.50 [113.31, 154.00] | 148.47 [124.42, 170.13] | <0.001 |
| Landmark of 3 days | |  |  |  |
| N | 2516 | 2242 | 274 |  |
| VMBG | 129.00 [113.00, 152.50] | 127.67 [112.23, 150.00] | 145.17 [125.04, 164.88] | <0.001 |
| FMBG | 133.33 [115.00, 154.69] | 131.61 [114.10, 152.89] | 146.14 [124.88, 166.69] | <0.001 |

**Table 5** Multivariate Cox regression analysis of VMBG, FMBG and 30-day mortality of the landmark cohorts

| **Models** | **HR(95%CI)** | **P** | **C-index** |  | **Brier** |
| --- | --- | --- | --- | --- | --- |
| Landmark at 2 days point |  |  |  |  |  |
| VMBG | 1.004(1.001,1.008) | 0.013 | 0.813 |  | 0.083 |
| FMBG | 1.004(1.001,1.008) | 0.027 | 0.813 |  | 0.083 |
| Landmark at 3 days point |  |  |  |  |  |
| VMBG | 1.006(1.003,1.010) | ＜0.001 | 0.812 |  | 0.080 |
| FMBG | 1.005(1.001,1.009) | 0.021 | 0.811 |  | 0.080 |

Adjusted for sex, age, race, comorbidity index, cerebrovascular disease, liver disease, chronic pulmonary disease, diabetes, congestive heart failure, cancer, renal disease, CRRT, ventilation, insulin, transfusion, SOFA, GCS, AKI stage, SAPSⅡ, APSⅢ, OASIS.

**Figure 1** Number of venous glucose measurements within 30 day (A). Number of fingertip glucose measurements within 30 day (B).

**Figure 2** Forest plots for subgroup analyses of VMBG and FMBG with 30-day mortality

**References**

[1] Lee SA, Cozzi M, Bush EL, Rabb H. Distant Organ Dysfunction in Acute Kidney Injury: A Review. Am J Kidney Dis. 2018;72(6):846-856. doi: 10.1053/j.ajkd.2018.03.028. PMID: 29866457.

[2] Kanji S, Buffie J, Hutton B, Bunting PS, Singh A, McDonald K, et al. Reliability of point-of-care testing for glucose measurement in critically ill adults. Crit Care Med. 2005;33(12):2778-85. doi: 10.1097/01.ccm.0000189939.10881.60. PMID: 16352960.

Once again, thank you very much for your comments and suggestions.

Yours sincerely,

Yun Liu
2026-02-02
